# The clinical performance of bulk-fill versus the incremental layered application of direct resin composite restorations: a systematic review

**DOI:** 10.1038/s41432-023-00905-4

**Published:** 2023-07-04

**Authors:** Arjita Sengupta, Olga Naka, Shamir B. Mehta, Subir Banerji

**Affiliations:** 1 Faculty of Dentistry Oral and Craniofacial Sciences, King’s College London, London, UK; 2 School of Dentistry, Aristotle University of Thessaloniki, 54124 Thessaloniki, Greece

**Keywords:** Bonded restorations, Composite resin

## Abstract

**Objectives:**

To systematically review the scientific evidence comparing the clinical effectiveness of bulk-fill versus incrementally layered conventional resin composites and to evaluate if one method offers clear merits with specific clinical outcomes.

**Materials and methods:**

Using relevant mesh terms and pre-established eligibility criteria in PubMed, Embase, Scopus and Web of Science, a thorough scientific search was conducted with an end-date of 30.04.2023. Randomized controlled clinical trials that involved the direct comparison of Class I and Class II resin composite restorations applied using incremental layering techniques versus bulk-filled in permanent teeth with an observation period of at least six months were considered. To evaluate the bias risk of the finalized records, a revised version of the Cochrane risk-of-bias tool for randomized trials was implemented.

**Results:**

Out of the 1445 records determined, 18 eligible reports were chosen for qualitative analysis. Data obtained was categorized as per, the cavity design, the intervention, the comparator(s), the methods of success/failure assessment, the outcomes, and follow-up. Two studies demonstrated an overall low-risk of bias, fourteen studies raised some concerns, and two studies exhibited high-risk.

**Conclusion:**

Bulk filled resin composite restorations demonstrated clinical outcomes similar to those of incrementally layered resin composite restorations within a review interval of 6 months to 10 years.

Key points

- Provides a comparison of the clinical efficacy of incremental layering versus bulk-fill techniques for the application of direct resin composites.
- Indicates bulk-fill technique as a good alternative to incremental layering.
- Suggests that the outcome of any restorative technique is dependent on patient, operator, material, cavity, and oral condition factors.

## Introduction

Resin composite has been the preferred direct restorative material by dental practitioners for the treatment of anterior and posterior teeth, with acceptable success rates and longer-term clinical performance reported in the literature^1^. Resin composite materials offer the benefits of ease of handling, superior physical properties, ability to polymerize on demand, availability in wide range of colors and translucencies, biocompatibility, and satisfactory adhesion to the dental hard tissues^2^. Composites have reported excellent clinical performance with annual failure rates ranging from 1% to 5% and 1% to 3% for anterior and posterior teeth respectively^3^. Furthermore, composite resin materials may offer the scope to allow repair^4^, strengthen the residual tooth structure, and their prescription may facilitate conservative cavity preparation^5^.

The main challenges encountered when using direct composite resins are, polymerization shrinkage and shrinkage stress, the degree of polymerization conversion, and their limited depth of cure. These factors may influence clinical performance. Adequate polymerization and the use of proper placement techniques are critical for the optimal clinical performance of these restorations^6^.

Incremental layering of composite resin using increments of ≤2 mm has been suggested to decrease shrinkage stress, improve the degree of conversion, evade disintegration of the restoration margin, and provide adequate esthetics^7^. The three-site method followed by the use of an oblique layering technique has been considered to be a good approach for composite layering technique and aid with the reduction of polymerization shrinkage^8^. The split, simultaneous modeling of separated increments has also been suggested to reduce shrinkage issues^9^. Lower levels of microleakage have been reported with the use of a split horizontal incremental technique at the gingival margin of a cavity^10^. At the occlusal margin of a Class II restoration, the application of a split horizontal incremental technique followed by the application of a centripetal and oblique placement technique has demonstrated the lowest levels of microleakage^11^.

The incremental application of resin composite is, however, time consuming. It can be challenging whilst restoring more conservative cavities and is associated with the increased risk of contamination. The incremental application technique also has the scope for unwanted air entrapment between successive layers, which may culminate in adhesive failure between layers^12^. A rise in the elastic modulus and post-photopolymerization shrinkage has been observed with increasing number of increments^7,13^.

The challenges with incremental layering have paved the development of bulk-fill composite materials which may be applied in layers of thickness of 4–5 mm, thereby offering the merit of reduced treatment time and the potential of reduced volumetric shrinkage stress as well as improved curing depth whilst maintaining the desired micromechanical properties^12^.

Polymerization shrinkage of bulk fills is decreased by incorporation of stress modulators like addition-fragmentation monomer (AFM), aromatic urethane dimethacrylate (AUDMA); high molecular mass monomers such as BisEMA, UDMA, BisGMA, Procrylat; and highly reactive photoinitiators. Initiator system optimization and the inclusion of fillers like zirconium / silica, ytterbium trifluoride, proacrylate, mixed oxides, and barium aluminum silicate particles in bulk fill resins have also improved their radiopacity and curing depth^14^. Polymerization depth is enhanced by better light transmission to deeper areas because of lowered light dispersion at the filler-matrix meeting point by reducing filler load, and/or improving filler particle size^15^.

Nevertheless, there is inconsistency in determining the curing depth in the literature and a concern among clinicians regarding the degree of conversion^16^. Furthermore, some constituents and modifications have been reported by the manufacturers of bulk-fill composites, but still certain constituents are unrevealed which may affect the ultimate clinical performance^14^.

There is a need for a new systematic review that helps clinicians understand the clinical effectiveness of the two composite placement techniques (incremental and bulk-fill). This will also enable practitioners to decide if they can select bulk technique as a reliable alternative method to incremental technique.

The aim of this review was to assess the efficacy of incremental layering versus bulk-fill techniques for the fabrication of direct composite restorations by evaluating their respective clinical outcomes for the restoration of permanent teeth. The objective was to systematically determine if one placement method offers clear benefits over the other by comparing their outcomes (success/failure) of clinical parameters like retention, recurrent/secondary caries, marginal discoloration/staining, marginal adaptation/integrity, fracture, postoperative sensitivity, anatomic form, color match, and surface texture/ roughness.

## Materials and methods

### The review protocol

The protocol was formulated considering the suggestions of the Cochrane Collaboration for systematic reviews and conforming with the Preferred Reporting Items for Systematic Reviews and Meta‐Analysis Protocols (PRISMA‐P) Statement recommendations^17,18^. This review was registered with the International Prospective Register of Systematic Reviews (PROSPERO) under the registration number CRD42021258095.

### Eligibility criteria

The *Review question* was, “In permanent teeth restored with direct composites, does incremental layering or bulk-fill technique perform better clinically?”

PICO model for clinical questions was used as follows:

*Participants-* Participants with permanent teeth restored using direct composite restorations.

*Intervention-* Bulk technique.

*Comparison-* Incremental layering technique.

*Outcomes-* Bulk-fill versus incrementally layered techniques were compared based on their performance (success/ failure) with regards to specific clinical parameters such as; retention, recurrent/secondary caries, marginal discoloration/staining, marginal adaptation/integrity, fracture, postoperative sensitivity, surface texture/ roughness, color match, and anatomical form.

*Types of studies-* Studies comparing Class I and Class II direct composite restorations restored by incremental layering technique versus bulk technique were involved. Randomized controlled trials (RCTs) were included in this review as bias is reduced by randomization and a meticulous instrument is provided to investigate the connection of an intervention and outcome by cause-effect association^19^. Non-randomized clinical studies, reviews, case reports, in vitro studies were excluded. Studies where a bulk fill composite was incrementally applied, or either bulk fill or incremental layering was individually assessed, were also excluded.

*Timing-* Studies with a review period of at least six months were included.

*Language-* Studies documented in English were selected.

*Publication status-* Only full papers published in peer-reviewed journals were considered.

### Information sources and search strategy

PubMed (National Centre for Biotechnology Information, NCBI)/ MEDLINE (National Library of Medicine), EMBASE (OVID interface), Scopus (Elsevier B.V.), and Web of Science were the electronic databases that were applied. Scientific articles were selected from the electronic databases using different combinations of text words and medical subject headings (MeSH) related to ‘bulk-fill’, ‘incremental layering’, ‘conventional composite’, and ‘direct restorations.’ The electronic probing was accompanied by manually searching of the Journal of Esthetic and Restorative Dentistry, Operative Dentistry, and the Journal of Conservative Dentistry. Furthermore, reference lists of the studies involved were scanned to confirm information saturation of pertinent studies. There was no restriction with regards to the publication date of the literature search. Before the final analysis, the search was repeated and carried out until 30.04.2023.

### Study selection

In RefWorks, “Close Duplicates” plus “Exact Duplicates” options were selected in the “View” tab and all associated citations were removed. The remaining studies were screened and evaluated as per their titles and abstracts. Trials that addressed the review question and met the eligibility benchmark, were shortlisted. Consequently, articles that met all the eligibility criteria were finalized. The whole process was provided by one researcher because the basis for this was a thesis project, and any concerns were discussed with the supervisors.

### Data extraction

A checklist of information was attained from the selected articles to provide relevant information. Data collected from the shortlisted studies were organized in the form of tables to enable the presentation and evaluation of the proof acquired.

### Evaluation of risk of bias in individual trials

The Oxford CEBM (Centre of Evidence-Based Medicine) tool was utilized to ascertain the level and grade of evidence of the articles involved in the review ranging from the highest Level 1a to the lowest Level 5.

The bias risk of the randomized clinical studies involved in the present systematic review were determined with the help of revised Cochrane risk-of-bias tool for randomized trials (RoB 2)^20^. If the Cochrane Handbook criteria was fulfilled by all the components, it was considered low bias risk; if the elements were questionable, it was viewed as high risk and, if inadequate attributes were found, it was marked as some concerns^17^.

## Results

### Selection of studies

**Fig. 1:**
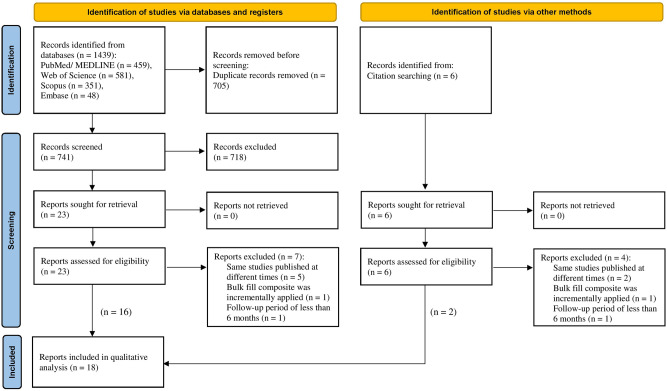
Flowchart showing the different stages of the systematic review.

As shown by Fig. 1, 1445 records were found following the primary investigation of the databases. The full text of 30 reports were thoroughly examined. Further eleven full-text reports were discarded^21–31^, and their exclusion criteria are listed in Table 1. Eighteen studies were evaluated for their study design and methods for final analysis of the results.

**Table 1:** Excluded studies with reasons.

Reasons for exclusion	Studies excluded
Same studies published at different times	*N* = 7^21–27^
Bulk fill composite was incrementally applied	*N* = 2^28,29^
Follow-up period of less than 6 months	*N* = 2^30,31^

### Quality assessment

All the included records displayed level 1b evidence [Individual randomized controlled trials (with narrow confidence interval)]. Since, consistent level 1 studies were observed, the Grade of Recommendation as per CEBM is A.

### Characteristics of included studies

Descriptive information obtained in this systematic review is shown in Tables 2–4.

**Table 2:** Details of the study design and population of involved trials.

Study	Study design	Participants (M/ F)	Average age (range in years)	Teeth restored	Follow-up
Salem et al., 2022^36^	RCT double-blind, parallel	36 (9/27)	31 ± 7.6 years	36	1 year
Endo Hoshino et al., 2022^34^	RCT double-blind, split mouth	53	48.3 years (±10.0)	159	4 years
Sekundo et al., 2022^37^	RCT double-blind, split mouth	60 (31/29)	(≥18)	120	3 years
Hardan et al., 2021^6^	RCT split mouth	30 (12/18)	25.8 ± 7.49 (18–45)	60	1 year
Durão et al., 2021^32^	RCT double-blind, split mouth	46 (22/24)	14.82 (12–18)	138	3 years
Balkaya & Arslan, 2020^38^	RCT double-blind, split mouth	54 (23/31)	22 (20–32)	109	2 years
Frascino et al., 2020^33^	RCT double-blind, split mouth	53	48.3 ± 10	159	1 year
Al-Sheikh, 2019^35^	RCT split mouth	40	(20–40)	80	6 months
Heck et al., 2018^39^	RCT split mouth	46	(≥18)	96	10 years
Atabek et al., 2017^40^	RCT split mouth	30	(7–16)	60	2 years
Bayraktar et al., 2017^41^	RCT split mouth	50	25.8 ± 7.49 (18–45)	200	1 year
Colak et al., 2017^42^	RCT double-blind, split mouth	34 (24/10)	33.74 ± 6.8 (23–56)	74	1 year
Karaman et al., 2017^43^	RCT double-blind, split mouth	37 (16/21)	27 (19–41)	94	3 years
Van Dijken & Pallesen, 2017^44^	RCT double-blind, split mouth	38 (22/16)	55.3 (32–87)	106	6 years
Yazici et al., 2017^45^	RCT double-blind, split mouth	50 (24/26)	(24–55)	104	3 years
Alkurdi & Abboud, 2016^46^	RCT parallel	60	(20–50)	60	1 year
Van Dijken & Pallesen, 2016^47^	RCT double-blind, split mouth	86 (44/42)	52.4 (20–86)	200	5 years
Manhart et al., 2010^48^	RCT split mouth	43	44.3 (19–67)	96	4 years

*RCT* randomized clinical trial.

**Table 3:** Comparative compendium of the included research articles.

Study	Type of Teeth and Restoration	Groups	Teeth Per Group	Etching Method	Adhesive System	Resin	Placement Technique
Salem et al., 2022^36^	Molar	Bulk Resin + Conventional Resin	18	Ultra-Etch selective enamel etching and rinsing	G-premio Bond	EverX posterior + G-ænial Sculpt	Two-step Bulk
	(Class II)	Conventional Resin	18			G-ænial Sculpt	Incremental
Endo Hoshino et al., 2022^34^	Premolar (94) and Molar (65)	Bulk Resin + Conventional Resin	53	35% phosphoric acid gel (Ultra-etch) etching and rinsing	Adper Single Bond 2	Filtek Bulk Fill Flow + Filtek Z350XT	Two-step Bulk
	(Class II)	Bulk Resin + Conventional Resin	53		XP Bond2	SureFil SDR + TPH3	Two-step Bulk
		Conventional Resin	53		Peak Universal	Amelogen Plus	Incremental
Sekundo et al., 2022^37^	Premolar (56) and Molar (64)	Bulk resin	60	35% phosphoric acid (Scotchbond Universal Etchant) selective enamel etching and rinsing	3M Scotchbond Universal adhesive	Filtek Bulk Fill Posterior	Bulk
	(Class II)	Conventional resin	60			Filtek Supreme XTE	Incremental
Hardan et al., 2021^6^	Premolar and Molar	Bulk resin	30	32% phosphoric acid gel (Scotchbond universal) etching and rinsing	Two-step etch-and-rinse Adper Single Bond	Filtek bulk-fill Posterior Restorative	Bulk
	(Class I)	Conventional resin	30			Filtek Z250XT	Incremental
Durão et al., 2021^32^	Premolar (46) and Molar (92)	Bulk resin	46	37% phosphoric acid selective enamel etching and rinsing	Clearfil SE Bond	Tetric EvoCeram bulk-fill	Bulk
	(Class I [101] and Class II [37])	Bulk resin	46			Filtek Bulk Fill	Bulk
		Conventional resin	46			Filtek Z250 XT	Incremental
Balkaya & Arslan, 2020^38^	Premolar (51) and Molar (58)	Bulk resin	38	Self-etch	Single Bond Universal adhesive	Filtek Bulk Fill Posterior Restorative	Bulk
	(Class II)	Conventional resin	37			Charisma Smart Composite	Incremental
Frascino et al., 2020^33^	Premolar (94) and Molar (65)	Bulk Resin + Conventional Resin	53	35% phosphoric acid gel etching and rinsing	XP Bond	SureFil SDR + TPH3	Two-step Bulk (4 mm + 2 mm)
	(Class II)	Bulk Resin + Conventional Resin	53		Adper Single Bond 2	Filtek Bulk Fill Flow + Filtek Z350XT	Two-step Bulk (4 mm + 2 mm)
		Conventional Resin	53		Peak Universal	Amelogen Plus	Incremental
Al-Sheikh, 2019^35^	Molar	Bulk resin	40	Tetric N-Etch etching and rinsing	Tetric N-Bond Total-Etch	Tetric EvoCeram bulk-fill	Bulk
	(Class I)	Conventional resin	40			Tetric EvoCeram	Incremental
Heck et al., 2018^39^	Molar	Bulk resin	46	Self-etch	Xeno III	QuiXfil	Bulk
	(Class II [82] and Class I [14])	Conventional resin	50	37% phosphoric acid etching and rinsing	Syntac classic	Tetric N-Ceram	Incremental
Atabek et al., 2017^40^	Molar	Bulk resin	30	Self-etch	OptiBond All-In-One	SonicFill	Bulk with sonic activation
	(Class I)	Conventional resin	30			Herculite Ultra	Incremental
Bayraktar et al., 2017^41^	Premolar (95) and Molar (105)	Bulk resin	50	Self-etch	AdheSE Bond	Tetric EvoCeram Bulk-Fill	Bulk
	(Class II)	Bulk resin+ Conventional resin	50		Single Bond Universal	Filtek Bulk-Fill Flowable +Filtek P60	Two-step Bulk (4 mm + 2mm)
		Bulk resin	50		OptiBond All-In-One	SonicFill	Bulk with sonic activation
		Conventional resin	50		Clearfil SE Bond	Clearfil Photo Posterior	Incremental
Colak et al., 2017^42^	Premolar (24) and Molar (50)	Bulk resin	37	Self-etch	AdheSE Bond	Tetric EvoCeram bulk-fill	Bulk
	(Class II)	Conventional resin	37			Tetric EvoCeram	Incremental
Karaman et al., 2017^43^	Premolar (41) and Molar (53)	Bulk resin + Conventional resin	47	35% phosphoric acid gel etching and rinsing	Adper Single Bond 2	x-tra base + GrandioSO	Two-step Bulk (4 mm+ 2 mm)
	(Class II)	Conventional resin	47			Aelite Flo + GrandioSO	Incremental
Van Dijken & Pallesen, 2017^44^	Premolar (47) and Molar (59)	Bulk resin + Conventional resin	53	Self-etch	XenoV	SDR flowable + Ceram X mono	Two-step Bulk (4 mm+ 2 mm)
	(Class I and Class II)	Conventional resin	53			Ceram X mono	Incremental
Yazici et al., 2017^45^	Premolar (54) and Molar (50)	Bulk resin	52	Etching and rinsing	Excite F	Tetric EvoCeram Bulk Fill	Bulk
	(Class II)	Conventional resin	52		Adper Single Bond 2	Filtek Ultimate	Incremental
Alkurdi & Abboud, 2016^46^	Premolar and Molar	Bulk resin	20	37% phosphoric acid etching and rinsing	Tetric N-Bond	Tertic N Ceram Bulk Fill	Bulk
	(Class II)	Bulk resin	20			SonicFill	Bulk with sonic activation
		Conventional resin	20			Tetric Evo Ceram	Incremental
Van Dijken & Pallesen, 2016^47^	Premolar (67) and Molar (133)	Bulk Resin + Conventional resin	100	Self-etch	XenoV	Ceram X mono	Incremental
	(Class II [124] and Class I [76])	Conventional Resin	100			SDR flowable + Ceram X mono	Two-step Bulk (4 mm + 2 mm)
Manhart et al., 2010^48^	Molar	Bulk resin	46	37% phosphoric acid etching and rinsing	Xeno III	QuiXfil	Bulk
	(Class II and Class I)	Conventional resin	50		Syntac classic	Tetric Ceram	Incremental

**Table 4:** Summary of the outcomes of placement techniques applied in the included studies.

Study	Assessment criteria	Incremental layering technique failure(s) and (total remaining)	Bulk fill technique failure(s) and (total remaining)
Salem et al., 2022^36^	Modified US Public Health Service	– (Total = 16)	– (Total = 18)
Endo Hoshino et al., 2022^34^	Modified US Public Health Service	Retention (*n* = 5) Marginal integrity (*n* = 1) Secondary caries (*n* = 1) (Total = 37)	**Filtek Bulk Fill Flow + Filtek Z350XT** Retention (*n* = 4) Marginal integrity (*n* = 2) Marginal discolouration (*n* = 2) Secondary caries (*n* = 1) (Total = 35) **SureFil SDR + TPH3** Retention (*n* = 3) Marginal integrity (*n* = 1) Marginal discolouration (*n* = 1) Secondary caries (*n* = 2) (Total = 34)
Sekundo et al., 2022^37^	Modified FDI World Dental Federation (esthetic, functional and biological properties)	***(Biological)*** Recurrent caries (*n* = 1) ***(Functional)*** Tooth fracture (*n* = 1) Restoration fracture (*n* = 1) Retention (*n* = 2) (Total = 49)	***(Biological)*** Recurrent caries (*n* = 1) ***(Functional)*** Tooth fracture (*n* = 1) Restoration fracture (*n* = 1) (Total = 48)
Hardan et al., 2021^6^	FDI World Dental Federation (esthetic, functional and biological properties)	***(Biological)*** Sensitivity (*n* = 4) ***(Esthetic)*** Marginal Discoloration (*n* = 4) ***(Functional)*** Marginal Adaptation (*n* = 1) (Total = 30)	***(Biological)*** Sensitivity (*n* = 2) ***(Esthetic)*** Marginal Discoloration (*n* = 2) (Total = 30)
Durão et al., 2021^32^	Modified US Public Health Service FDI World Dental Federation (esthetic, functional and biological properties)	– (Total = 36) ***(Biological)*** Postoperative sensitivity (*n* = 2)	**Tetric EvoCeram bulk-fill-** Marginal adaptation (*n* = 1), Recurrent caries (*n* = 1) (Total = 36) **Filtek Bulk Fill-** 0 (Total = 36) **Tetric EvoCeram bulk-fill-** ***(Biological)*** Recurrent caries (*n* = 1)
Balkaya & Arslan, 2020^38^	Modified US Public Health Service	– (Total = 32)	– (Total = 31)
Frascino et al., 2020^33^	Modified US Public Health Service	– (Total = 53)	- (Total = 53 in each group)
Al-Sheikh, 2019^35^	Modified US Public Health Service	Retention (*n* = 1) (Total = 37)	Retention (*n* = 2) (Total = 37)
Heck et al., 2018^39^	Modified US Public Health Service	Secondary caries (*n* = 2) [FDI tooth no.16, 37], Marginal integrity (*n* = 1) [FDI tooth no.16], Tooth fracture (*n* = 1) [FDI tooth no. 26] (Total = 30)	Secondary caries (*n* = 2) [FDI tooth no. 27, 36], Postoperative sensitivity (*n* = 1) [FDI tooth no.36], Restoration fracture (*n* = 1) [FDI tooth no.36], Tooth fracture (*n* = 1) [FDI tooth no.36] (Total = 26)
Atabek et al., 2017^40^	Modified US Public Health Service	– (Total = 30)	– (Total = 30)
Bayraktar et al., 2017^41^	Modified US Public Health Service	Secondary caries (*n* = 1) (Total = 43)	**Tetric EvoCeram bulk fill-** Secondary caries (*n* = 2), Marginal integrity (*n* = 1), Anatomic form (*n* = 1) **Filtek bulk fill flowable + Filtek P60 –** Retention (*n* = 1), Postoperative sensitivity (*n* = 1), Secondary caries (*n* = 2), Marginal adaptation (*n* = 2), Anatomic form (*n* = 2) **Sonic Fill-** 0 (Total = 43 in each group)
Colak et al., 2017^42^	Modified US Public Health Service	Marginal discoloration (*n* = 1) (Total = 35)	– (Total = 35)
Karaman et al., 2017^43^	Modified US Public Health Service	– (Total = 33)	– (Total = 33)
Van Dijken & Pallesen, 2017^44^	Modified US Public Health Service	Fracture of resin composite (*n* = 2), Tooth fracture (*n* = 1) (Total = 49)	Fracture of resin composite (*n* = 2), Secondary caries (*n* = 1) (Total = 49)
Yazici et al., 2017^45^	Modified US Public Health Service	- (Total = 40)	– (Total = 41)
Alkurdi & Abboud, 2016^46^	Modified US Public Health Service	Marginal discoloration (*n* = 1) (Total = 19)	**Sonic fill-** 0 (Total = 20) **Tetric N-Ceram bulk fill-** Postoperative sensitivity (*n* = 2), Marginal discoloration (*n* = 2) (Total = 19)
Van Dijken & Pallesen, 2016^47^	Modified US Public Health Service	Secondary caries (*n* = 1), Caries and tooth fracture (*n* = 1), Tooth fracture (*n* = 2), Fracture of resin composite (*n* = 2) (Total = 91)	Secondary caries (*n* = 1), Caries and tooth fracture (*n* = 1), Tooth fracture (*n* = 2) (Total = 92)
Manhart et al., 2010^48^	Modified US Public Health Service	Tooth fracture (*n* = 1) [FDI tooth no. 26] (Total = 46)	Postoperative Sensitivity (*n* = 1) [FDI tooth no. 36], Tooth fracture (*n* = 2) [FDI tooth no. 36, 47], Fracture of resin composite (*n* = 1) [FDI tooth no.36] (Total = 37)
		Failures (*n* = 41) (Total restorations = 706)	Failures (*n* = 59) (Total restorations = 922)

### Assessment of risk of bias

**Fig. 2:**
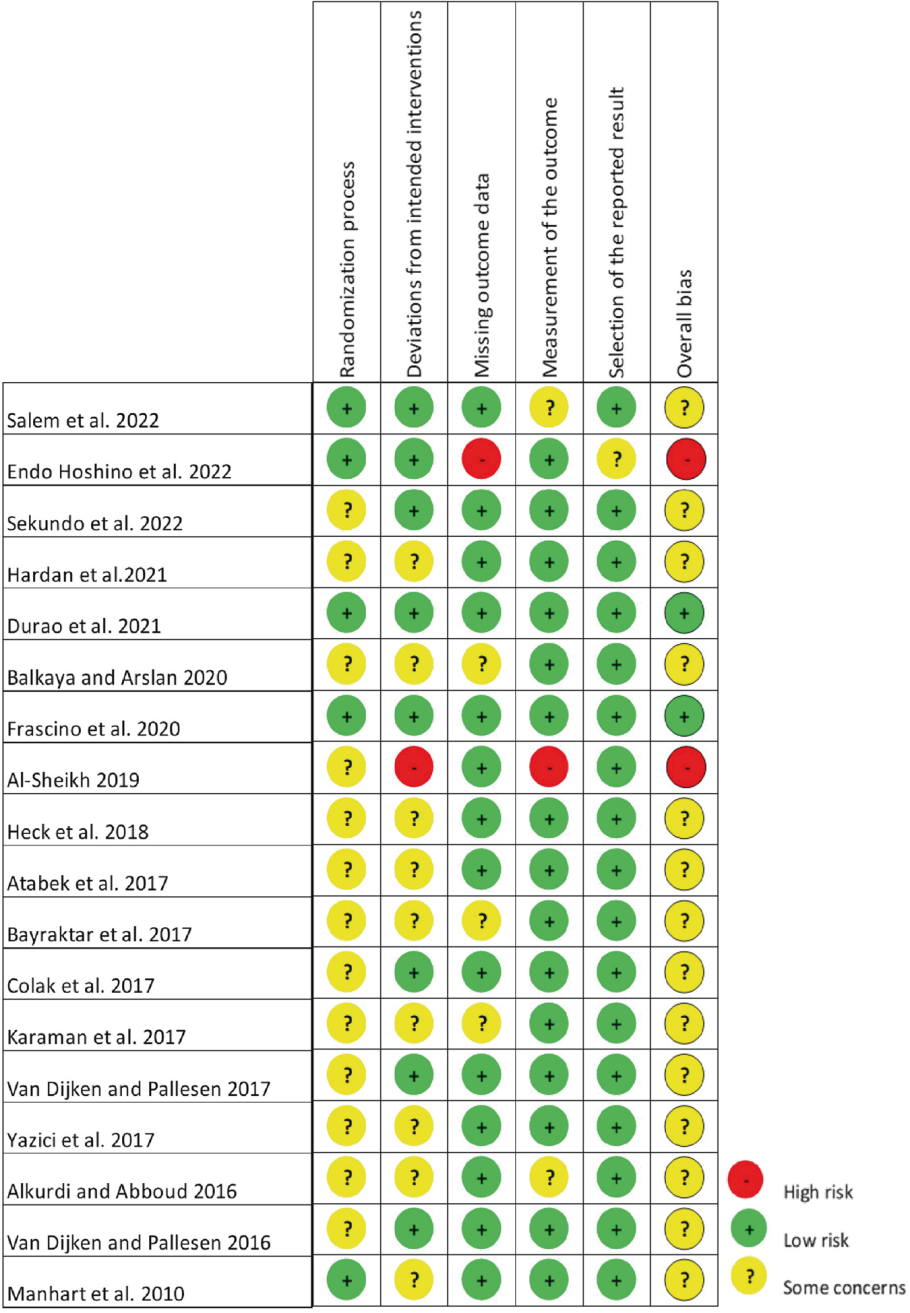
Cochrane-style risk of bias figures, which display the domain and overall judgements study-by-study.

**Fig. 3:**
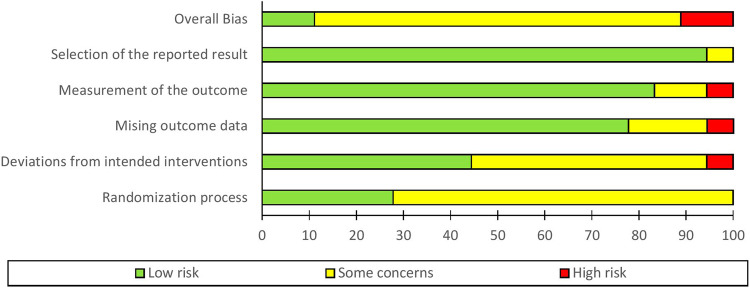
Bar graphs of risk of bias.

Overall, bias risk for all domains was low for two studies^32,33^ (11.1%). Two studies^34,35^ demonstrated high risk (11.1%); and fourteen studies^6,36–48^ showed some concerns (77.8%) for overall bias risk as shown in Figs. 2 and 3.

### Synthesis of results

Due to the variety of composite materials and bonding systems used, and the differences in the design of the involved studies, a meta-analysis that requires relatively homogenous studies could not be performed. Thus, the results were summarized in a qualitative way by listing data from the included studies as per the cavity design, the intervention, the comparator(s), the methods of success/failure assessment, the outcomes, and follow-up. The quality of the data obtained from the included studies was weighed via the risk of bias assessment, giving greater reliability to the higher quality study results.

### Analysis of the systematic reviews with a relevant review question

A 16 item-criterion appraisal tool known as AMSTAR-2 was applied to methodologically inspect the quality of systematic reviews with a relevant review question^49^. The evaluation of the systematic reviews by AMSTAR 2 (Table 5) graded one study^50^ moderate quality as no critical flaws were found but study design selection was not explained, the effect of risk-of-bias (RoB) from included studies on the outcome of meta-analysis was not assessed, and funding sources for involved studies was not reported. Two studies^51,52^ were graded critically low, as both had critical flaws such as review protocol was not registered before commencement and the publication bias was not assessed. Additionally, exclusion of individual studies was not justified in one study^52^.

**Table 5:** Evaluation of systematic reviews with AMSTAR-2.

	Arbildo-Vega et al. (2020)^51^	Cidreira Boaro et al. (2019)^52^	Veloso et al. (2019)^50^
1. In the research questions and inclusion criteria, PICO components were included	Yes	Yes	Yes
2. Before commencement of review, protocol was registered, and any crucial deviations were justified (CRITICAL)	No	No	Yes
3. The study design selection for inclusion was explained by review authors	No	No	No
4. Literature search strategy used by review authors was comprehensive (CRITICAL)	Partial yes	Partial yes	Partial yes
5. The study selection was performed in duplicate	Yes	Yes	Yes
6. The extraction of data was performed in duplicate	Yes	No	Yes
7. The excluded studies were listed, and its rationale was provided (CRITICAL)	Yes	No	Yes
8. The involved studies were described in adequate detail	Yes	No	Yes
9. In individual studies involved in the review, the risk of bias (RoB) was evaluated using a satisfactory technique (CRITICAL)	Partial yes	Partial yes	Partial yes
10. Funding sources for included studies was reported	No	No	No
11. Appropriate methods were used for the meta-analysis performed (CRITICAL)	Yes	Yes	Yes
12. On the outcome of meta-analysis or other evidence synthesis, the possible impact of RoB was assessed in individual studies	No	No	No
13. When explaining the outcome of the review, RoB was considered for individual studies (CRITICAL)	Yes	Yes	Yes
14. Any variability noticed in the outcome of the review was satisfactorily explained and discussed	Yes	Yes	Yes
15. Publication bias was adequately investigated and its impact on the results was discussed in quantitative synthesis (CRITICAL)	No	No	Yes
16. For conducting the review, any funding was received or potential sources of conflict of interest was reported	Yes	Yes	Yes

## Discussion

Traditionally, the incremental application of resin composite is prescribed to allow efficient polymerization, to control polymerization shrinkage and stress, and to improve the C-factor. However, aggravated deformation of compromised cusps has been demonstrated with increasing number of increments^13^. Also, when incrementally layering composites, void formation between increments can take place, resulting in sensitivity, deterioration of the resin material, and cuspal deflection^53,54^. On the other hand, the bulk-fill application technique provides ease of placement, lower technical sensitivity, and is less time-consuming^31^. Bulk-filling also prevents disintegration of mechanical properties, with a reduced risk of void formation^55^. But, there is a significant variation between individual bulk-fill products regarding filler particle size and framework, plus method of clinical placement, which can impact their clinical performance^14^.

According to the studies included in this review, bulk-fill technique demonstrated no notable distinction in clinical performance compared to the incremental layering technique for the specific clinical parameters considered.

The success of a restoration in clinical studies is demonstrated by its endurance in the oral cavity, making retention the most significant evaluation criteria^42^. One report recorded retention failure with two Tetric EvoCeram bulk-fills and one incrementally placed Tetric EvoCeram restoration. The difference in retention was linked to the adhesive material or the method used^35^. Loss of retention was recorded with two Filtek Supreme XTE and none with Filtek Bulk Fill Posterior in a three-year study^37^. This was associated with the viscoelastic property of the bulk-fill material used due to which shrinkage stress was not a problem^56^. One Filtek bulk-fill was lost in a study due to technical error during restoration placement^41^.

Fracture and recurrent caries formation are the primary causes for the failure of directly placed composite restorations^1,57^. Even in this systematic review, the reasons for failures predominantly were tooth- and resin-fractures, followed by secondary caries in both bulk-fill restorations and incrementally layered conventional restorations respectively. Patients with temporomandibular disorders or parafunctional habits like bruxism can eventually impact the sound tooth, resulting in restoration- and tooth-fractures^3,58^. Two studies demonstrated a substantially notable number of failures caused by resin composite and/or tooth fractures, mostly amongst bruxist patients^44,47^. One report recorded teeth fractures with Quixfil bulk restorations (*n* = 2) and incremental Tetric Ceram resin (*n* = 1)^48^. Fracture risk is higher when premature fatigue of the bonding agent occurs at the restoration-tooth interface^57,59^, which was considered by the study as the probable cause of failure with Quixfil bulk-fills (*n* = 3)^48^. High fracture rate of resin composites, both conventional and bulk composites has been reported by many included studies^37,39,44,47,48^, which could be a material-specific constrain of composites^3^.

Biological factors may be the cause of secondary caries instead of the material being used to restore^60^. High caries-risk individuals or low socio-economic status patients with restricted access to health services are susceptible to recurrent caries formation^1,57,61^. In this review, patients with substandard dental hygiene and high caries risk were excluded in several records^35–37,40–43,45,46,48^. However, two records included high-risk caries participants which was linked to SDR flowable bulk +Ceram X mono (*n* = 2) and incremental Ceram X mono (*n* = 1) restoration failures due to secondary caries^44,47^. Despite selecting patients with good dental hygiene, in one of the included trials, secondary caries was detected with incrementally placed Tetric N-Ceram resins (*n* = 2) and QuiXfil bulk-fills (*n* = 2). It was assumed to be connected to restorative material’s physical framework or the adhesive system efficacy^39^.

During restoration placement, contamination with saliva and marginal adaptation faults were associated with secondary caries development (*n* = 5) in another study^41,61,62^. According to an earlier systematic review, a lower failure rate of direct restorations was observed to occur with the use of rubber dam isolation than those performed using saliva ejectors and cotton rolls as a means of attaining the required moisture control^63^. Amongst the four studies in this review where rubber dam isolation was used, the absence of any restorative failures were observed^33,36,40,45^.

Marginal adaptation is affected by the long-term deterioration of the bonding system and polymerization shrinkage of the composite used^64^. Marginal adaptation may deteriorate over time by hydrolysis of the adhesive interface which occurs when monomers absorb water and chemicals^65^. This was seen in an included study where marginal integrity had declined after 10 years in both groups^39^. Hydrolytic degradation of Optibond All-In-One adhesive was documented by included records which might have influenced the adhesive strength of restorations, thereby affecting the marginal integrity^40,41^. Using radiological assessment, marginal adaptation was found to be good over time with Filtek bulk fill posterior restorative group, whereas a formation of gap was observed from first day in 96.7% of the incrementally layered Filtek Z250XT group^6^. The ISO requirements for radiopacity were met by Filtek bulk fill posterior restorative in this study^66^. The marginally higher radiopacity of the incremental nanohybrid than enamel may have caused accurate discernment of defects^60^.

Another study reported marginal degradation of incrementally packed Amelogen Plus restorations, beginning at six-months and deterioration at one-year follow-up. Conversely, the low shrinkage SureFil SDR bulk-fill and Filtek Bulk Fill Flow restorations showed marginal alteration only after a year^33^. Equivalent results were assessed in a similar study conducted with the same three resin composites^34^. This may be associated with the low elastic modulus of the bulk-fills, decreasing the polymerization stresses and hence, sustaining the marginal adaptation^67^. Likewise another record demonstrated poor marginal integrity of incrementally layered Filtek Ultimate restorations in comparison to Tetric EvoCeram Bulk Fill restorations^45^, caused by the increased water sorption of low molecular-weight monomers with the former^68^ and lower polymerization shrinkage of the latter^69^. Corroborating with a systematic review and meta-analysis of in vitro studies^70^, conventional resin composites with incremental techniques were found to have marginal integrity comparable to bulk fill composites in an included report^34^.

Marginal staining may be the first clinical sign of restoration failure^71^. It is usually caused by the faults present between the cavity margins and composite restoration because of substandard bonding, ineffective composite placement, or polishing methods, and/ or by successive stress fatigue^72–75^. Higher marginal discolouration may be linked to the presence of poor marginal adaptation^76^. The same was noted in an included study with incremental Filtek Ultimate group where marginal defects from contraction stress might have produced staining^45^. Marginal staining was reported in another report with multi-layered Tetric EvoCeram (*n* = 1) and Tertic N-Ceram Bulk Fill (*n* = 2). In comparison, no failures were seen with Sonic fill composite resin whose viscosity is reduced due to sonic vibration, resulting in better adaptation to the cavity walls and hence, improved marginal properties^46^.

Marginal discolouration has been documented to occur more frequently in cases using the self-etch technique^77–79^. According to a study, low bravo scores for marginal discoloration of both nano-hybrid (Tetric EvoCeram) and Tetric EvoCeram bulk-fill restorations may be because etching with phosphoric acid was not done^42^. Significantly lower marginal discolouration was observed with the restoration of cavities with bulk-fill composite in a single layer, compared to conventional composites in two included studies. But this was disregarded by both the studies as no additional treatment was required for minor surface discoloration^6,45^. Conversely, higher marginal discolouration was presented by bulk-fill composite systems than the conventional one in a study of this systematic review^34^.

Majority of the included studies recorded no post-operative sensitivity in the teeth restored^33–38,40,42–45,47^. Depth and dimensions of the cavity prepared, marginal seal and liner application in deep cavities, can also influence postoperative sensitivity^41,42,80–83^. Most studies assessing postoperative sensitivity did not describe the cavity depth or involved more shallow cavities. Only one study proved all the cavities being restored were 4–5 mm deep by radiographic assessment^40^ and most cavities were described to be deep in two studies^44,47^. Application of liners in deep cavities shields the pulpo-dentin complex, reducing heat/electric stimuli, dentin sensitivity, and helps in reparative dentin formation^84^. No post-operative sensitivity was noted in studies with liner applied in deep cavities^38,41,42^. Following 12-months, sensitivity was recorded in only one tooth with a deep cavity among the ones filled with bulk-fill via sonic activation. This was regarded to be caused by the absence of a calcium hydroxide based liner^40^. Use of flowable composite linings reported no restorative failures in another study^43^. Contradictory evidence was found concerning the application of liners and post-operative sensitivity in a Cochrane review^85^. According to one report, higher sensitivity was recorded with the use of incrementally applied Filtek Z250XT compared to Filtek bulk-fill posterior restorative, which may be linked to adhesive failure or cusp deflection^6^.

Surface texture modifications can be associated with the composite’s filler load, size, or hardness^86^. In one study, surface texture was rougher after finishing and polishing of some nanohybrid restorations, which was attributed to large fillers exfoliating from the matrix while polishing^87^. Slightly rougher surface was reported in a study with Filtek Ultimate group which was associated with void entrapment in the incremental layering method^45^. The differences in surface roughness / texture may be related to the fact that there is no specific finishing and polishing system for bulk-fill materials. For full-body bulk-fill resin composites, multistep finishing/polishing systems have been suggested to give greater polishability^88^.

Color stability in a study^35^ was contemplated to be influenced by intrinsic factors like resin’s organic matrix^86,89^. One trial reported better performance of incremental microhybrid Amelogen Plus compared to Filtek Bulk Fill Flow + Filtek Z350XT with regards to superficial staining^33^. This may be associated to better sorption ability of the nanoparticles in Filtek bulk fill^90^. Best results were observed with SDR + TPH3 as it was less prone to liquid absorption which was linked to the absence of triethylene-glycol dimethacrylate in TPH3^91^. Good color stability was seen because of compact filler particles present in bulk- and conventional-resins used in a study^46^, and because of resistance to color modification provided by the presence of Urethane Dimethacrylate (UDMA) polymer matrix like that found in an earlier study^92^.

In one study, sonic fill was found to provide anatomically superior results compared to incremental Filtek bulk fill, Tetric EvoCeram bulk fill, and Clearfil photo posterior^41^.

### Limitations of the study

The comparative assessment of both the techniques was difficult, as the included studies involved in the review had a number of variables such as, different etching and bonding techniques for different restorative materials (incrementally layered conventional resin composites and bulk-fill) along with varied patient, operator, cavity, and oral condition factors. Evaluation criteria methods were non-standardized and analysis for the clinical parameters were not always explicitly provided.

### Suggestions for future study

The comparison between the two composite placement techniques will be more adequate with studies involving similar materials with fewer variable factors. Clinical trials with an extended observation period are required to attain stronger evidence and information regarding the performance of layering techniques clinically.

## Conclusion

This systematic review disclosed that:

- Direct resin composite restorations fabricated using incrementally layered techniques performed clinically just as well as those formed using bulk-fill technique in the permanent dentition.
- The placement techniques demonstrated no significant differences with regards to, retention, recurrent/secondary caries, marginal discoloration/staining, marginal adaptation/integrity, fracture, postoperative sensitivity, surface texture/ roughness, color match, and anatomical form.
- The bulk-fill technique is a good alternative treatment option to incremental filling, offering reduced restoration time and the scope for a reduction in feasible operator errors.
